# Supplementary material for: A Critical View of the Application of the APEX Software (Aqueous Photochemistry of Environmentally-Occurring Xenobiotics) to Predict Photoreaction Kinetics in Surface Freshwaters
Source: Molecules. 2019 Dec 18;25(1):9. doi: 10.3390/molecules25010009 (PMC7017383; doi:10.3390/molecules25010009)
Supplement: Supplementary file 1 [file molecules-25-00009-s001.zip › 3.2.4_gcc-4.4.0.zip!/3.2.4_gcc-4.4.0/3.2.4_gcc-4.4.0/doc/octave/html/interpreter/Diagonal-and-Permutation-Matrices.html]

Diagonal and Permutation Matrices - Untitled


Next: Sparse Matrices,
Previous: Nonlinear Equations,
Up: Top


---

## 20 Diagonal and Permutation Matrices

- Basic Usage: Creation and Manipulation of Diagonal and Permutation Matrices- Matrix Algebra: Linear Algebra with Diagonal and Permutation Matrices- Function Support: Functions That Are Aware of These Matrices- Example Codes: Some Examples of Usage- Zeros Treatment: The Differences in Treatment of Zero Elements
